# Supplementary material for: Identification of a Zeb1 expressing basal stem cell subpopulation in the prostate
Source: Nat Commun. 2020 Feb 5;11:706. doi: 10.1038/s41467-020-14296-y (PMC7002669; doi:10.1038/s41467-020-14296-y)
Supplement: Supplementary file 1 — Supplementary Information [file 41467_2020_14296_MOESM1_ESM.pdf]

SUPPLEMENTARY INFORMATION

Identification of a Zeb1 expressing basal stem cell subpopulation in the prostate

by Xue Wang *et al*

Contents:

Supplementary Figure 1 | Analyses of Zeb1$^+$ basal cell proliferation and total cell number at different developmental time points
Supplementary Figure 2 | Analyses of Zeb1$^+$ basal cell proliferation and total cell number at different time points in prostate regression and regeneration
Supplementary Figure 3 | Lineage tracing of Zeb1$^+$ prostate basal cells in prostate regeneration of adult mice
Supplementary Figure 4 | Expression analyses of previously reported stem cell surface markers in Zeb1$^+$ prostate basal cells
Supplementary Figure 5 | Murine Lineage$^-$ Sca-1$^+$ CD49f^{hi} prostate cells are collected for Single cell RNA-seq
Supplementary Figure 6 | Single cell RNA-seq data pre-processing followed by clustering
Supplementary Figure 7 | GSEA enrichment and the differentiation trajectory analyses showing that the cell cluster 7 possesses stem cell expression features
Supplementary Figure 8 | Slingshot predicts three lineage trajectories originated from C7
Supplementary Figure 9 | Wnt signaling pathway plays a positive role in the outgrowth of Zeb1+ prostate basal cells
Supplementary Figure 10 | Pathological evaluation and feature characterization for Hi-Myc mice
Supplementary Figure 11 | Zeb1$^+$ epithelial cells are found in both basal and luminal layer from PKO and TRAMP prostate tumor mouse models
Supplementary Figure 12 | Gating strategies applied for flow cytometry data
Supplementary Table 1: Primers for qRT-PCR, genotyping and knock out Zeb1
Supplementary Table 2: Antibodies used in this paper

Supplementary Figures

Supplementary Figure 1

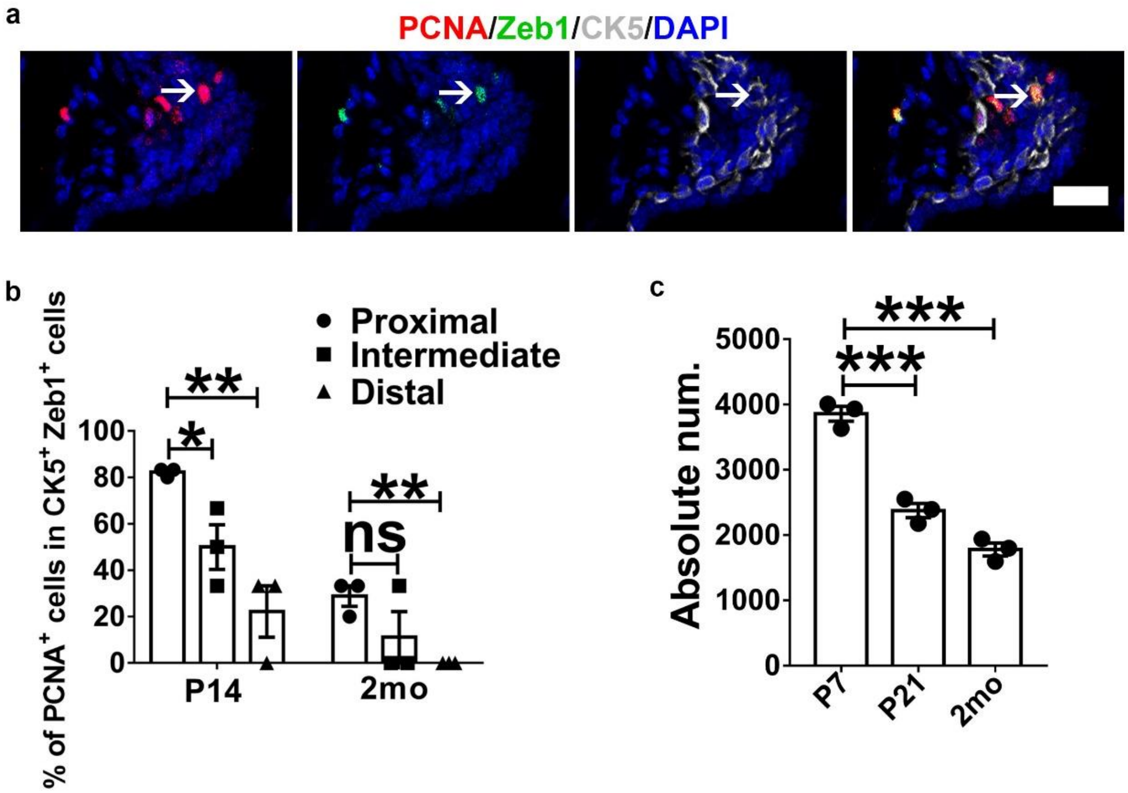

Supplementary Figure 1 | Analyses of Zeb1$^+$ basal cell proliferation and total cell number at different developmental time points.

(a) Images of PCNA, Zeb1 and CK5 triple staining on prostate sections from postnatal day 14 mice.

(b) Quantification of the percentage of proliferating cells in Zeb1$^+$ basal cells in urethral proximal, intermediate and distal regions at indicated developmental time points.

(c) Quantification of the absolute number of Zeb1$^+$ prostate basal cells at indicated developmental stages.

(n=3 mice. At least 20 fields per section of 3 sections each mouse prepared from 3 mouse prostates were analyzed. Data are analyzed by Student's t-test and are presented as mean + s.e.m. All scale bars = 20µm. *P<0.05, **P<0.01, ***P<0.001. Source data are provided as a Source Data file.)

Supplementary Figure 2

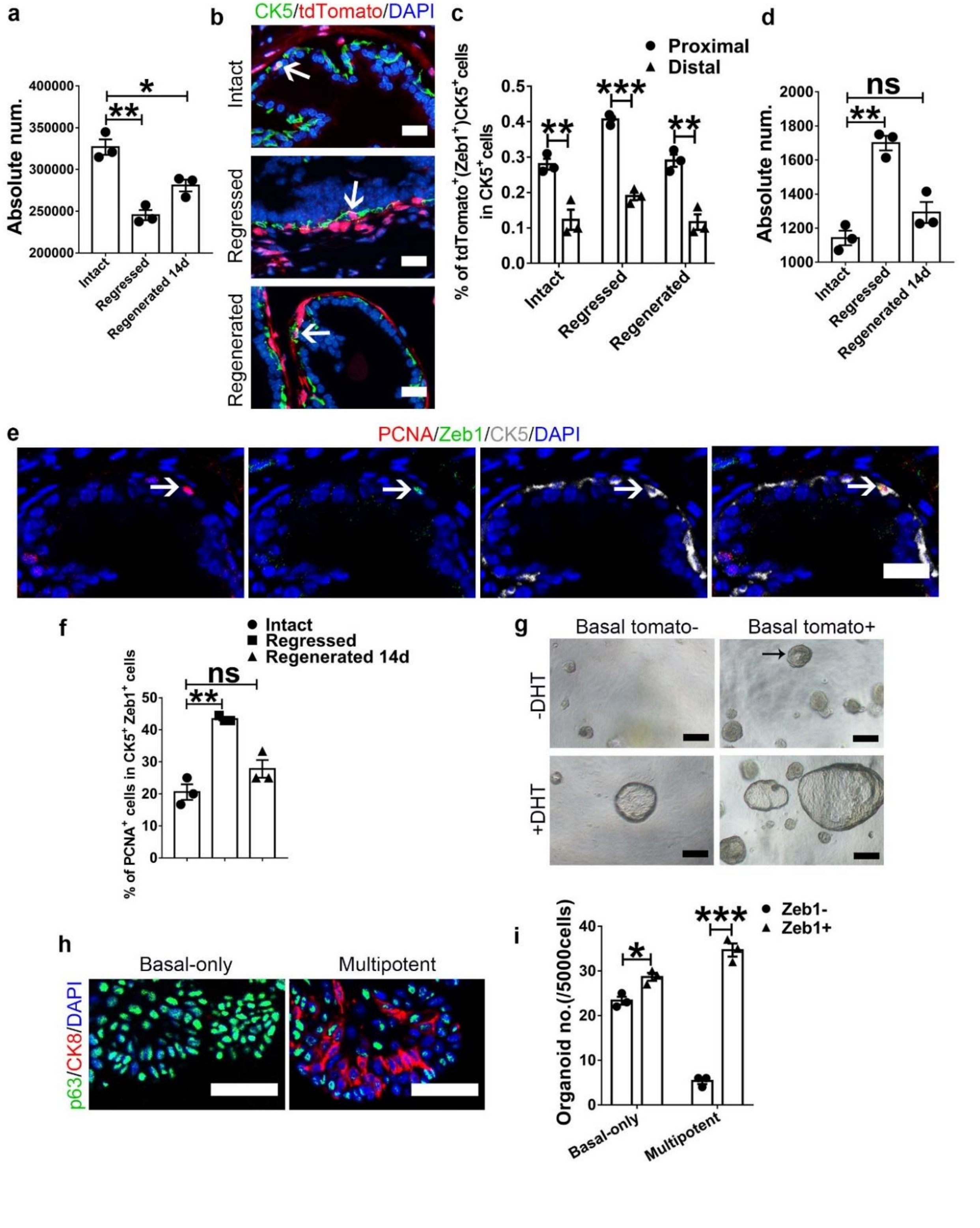

Supplementary Figure 2 | Analyses of Zeb1^{+} basal cell proliferation and total cell number at different time points in prostate regression and regeneration.

(a) Quantification of the absolute number of CK5^{+} prostate basal cells in intact, regressed and regenerated prostates. (n=3 mice. Data are analyzed by Student's t-test and are presented as mean + s.e.m. *P<0.05, **P<0.01. Source data are provided as a Source Data file.)

(b) Fluorescent immunostaining images of prostate sections from intact, regressed (21 days after castration) and regenerated (14 days after androgen replacement) prostate sections from Zeb1-tdTomato reporter mice. (Scale bars= 20μm)

(c) Quantification of the percentage of tdTomato (Zeb1)$^{+}$ cells in prostate basal cells at urethral-proximal or distal regions in intact, regressed and regenerated prostates. (n=3 mice. Data are analyzed by Student's t-test and are presented as mean + s.e.m. **P<0.01, ***P<0.001. Source data are provided as a Source Data file.)

(d) Quantification of the absolute number of Zeb1^{+} prostate basal cells in intact, regressed and regenerated prostates. (n=3 mice. Data are analyzed by Student's t-test and are presented as mean + s.e.m. **P<0.01. Source data are provided as a Source Data file.)

(e) Images of triple staining of PCNA, Zeb1 and CK5 on prostate sections from intact, regressed and regenerated mice. (Scale bars= 50μm)

(f) Quantification of the percentage of proliferating cells in total Zeb1^{+} basal cells at indicated time points of prostate regeneration. (n=3 mice. Data are analyzed by Student's t-test and are presented as mean + s.e.m. **P<0.01.)

(g) *In vitro* organoid-forming assay showing more organoids were produced from Zeb1+ basal cells with both basal-only and multipotent phenotypes compared to Zeb1- basal cells with androgen deprivation. (Scale bars= 200μm for bright-field images)

(h) Staining of p63 (green) and CK8 (red) on sections of organoids formed from basal epithelial cells cultured in the medium without androgen. (Scale bars= 20μm)

(i) Quantification of the number of basal-only or multipotent organoids formed from Zeb1^{+} or Zeb1^{-} prostate basal cells following androgen ablation. (n=3 replicates. Data are analyzed by Student's t-test and are presented as mean + s.e.m. *P<0.05, ***P<0.001. Source data are provided as a Source Data file.)

(At least 20 fields per section of 3 sections each mouse prepared from 3 mouse prostates were analyzed.)

Supplementary Figure 3

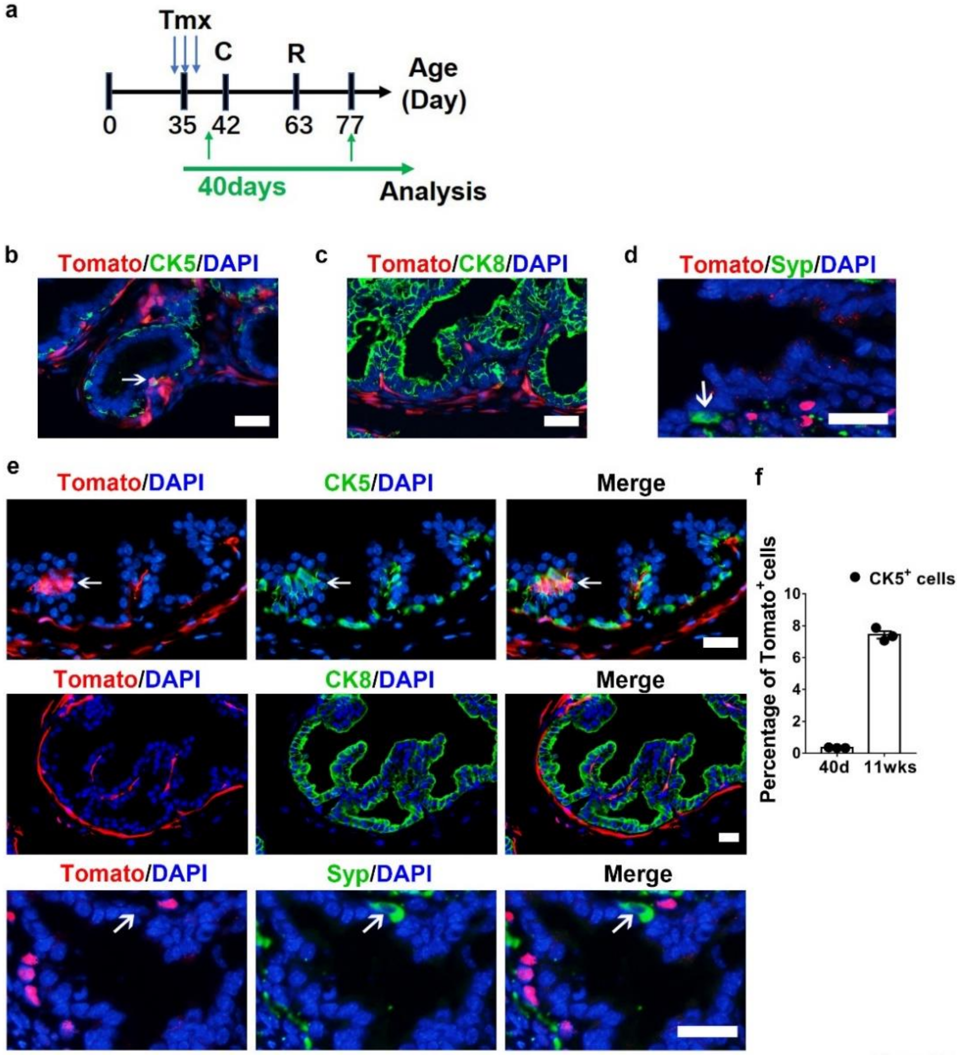

Supplementary Figure 3 | Lineage tracing of Zeb1$^+$ prostate basal cells in prostate regeneration of adult mice.

(a) Illustration of experiment procedure to trace the fate of Zeb1$^+$ basal cells during the adult prostate regeneration. **(b-d)** tdTomato is positively expressed in CK5$^+$ basal cells, but not in CK8$^+$ luminal cells or Syp$^+$ neuroendocrine cells from Zeb1-CreERT2/tdTomato mouse prostates at 2 days after tamoxifen administration. **(e)** Clusters of CK5$^+$ tdTomato$^+$ cells can be detected in the basal cell compartment but tdTomato can be barely found in CK8$^+$ or Syp$^+$ cells in the prostate. **(f)** Percentage of tdTomato$^+$ cells in CK5$^+$ cells in prostate from mice 2 days after tamoxifen injection and in regenerated prostates. (n=3 mice. Source data are provided as a Source Data file.)

(In this figure, at least 20 sections each mouse prepared from 3 mouse prostates were analyzed. At least 60 fields for each immunostaining experiment were collected for analysis. Data are analyzed by Student's t-test and are presented as mean + s.e.m. All scale bars = 25μm.)

Supplementary Figure 4

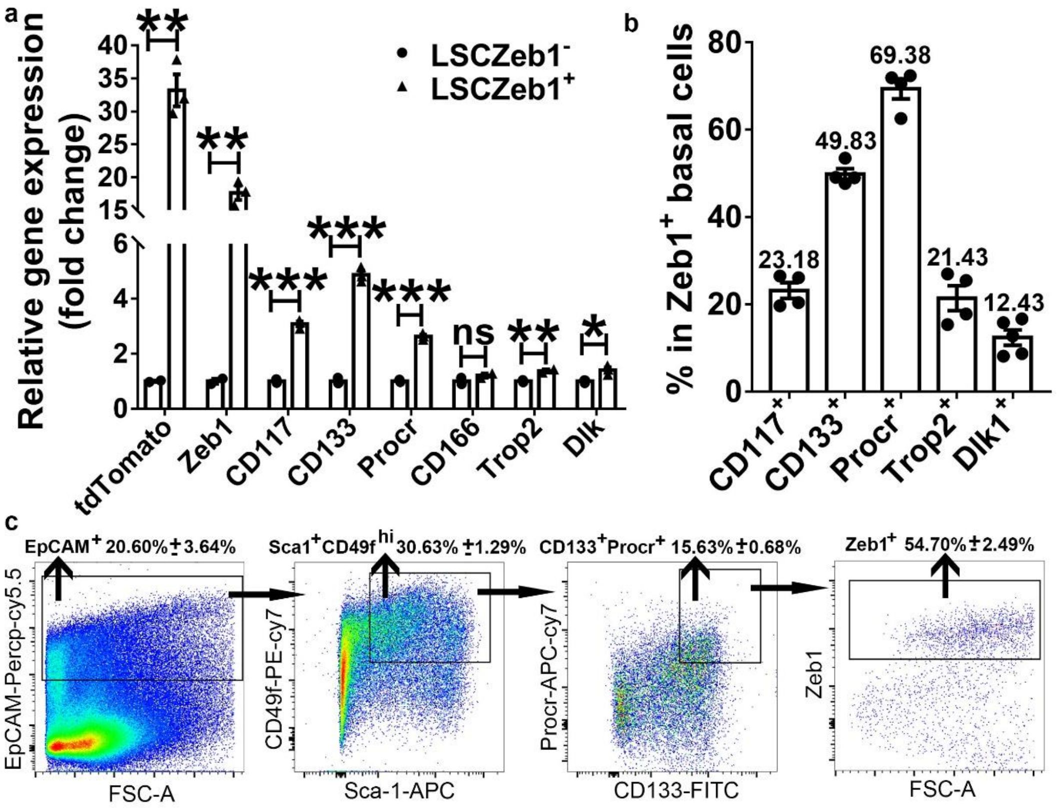

Supplementary Figure 4 | Expression analyses of previously reported stem cell surface markers in Zeb1⁺ prostate basal cells.

(a) qRT-PCR quantification of stem cell surface markers in sorted Tomato$^+$ (Zeb1$^+$) and Tomato$^+$ (Zeb1$^-$) prostate basal cells. RNA expression levels were normalized to Tomato$^+$ (Zeb1$^-$) prostate basal cells (n=3 independent experiments. Data are analyzed by Student's t-test and are presented as mean + s.e.m. *P<0.05, **P<0.01, ***P<0.001.)

(b) Flow cytometry analyses showing the percentage of previously reported stem cell surface markers in Zeb1$^+$ prostate basal cells. (n=4 mice.)

(c) FACS analyses displaying the percentage of Zeb1$^+$ basal cells in Epcam$^+$ Sca1$^+$ CD49f$^+$ CD133$^+$ Procr$^+$ basal cells. (n=5 mice.)

Supplementary Figure 5

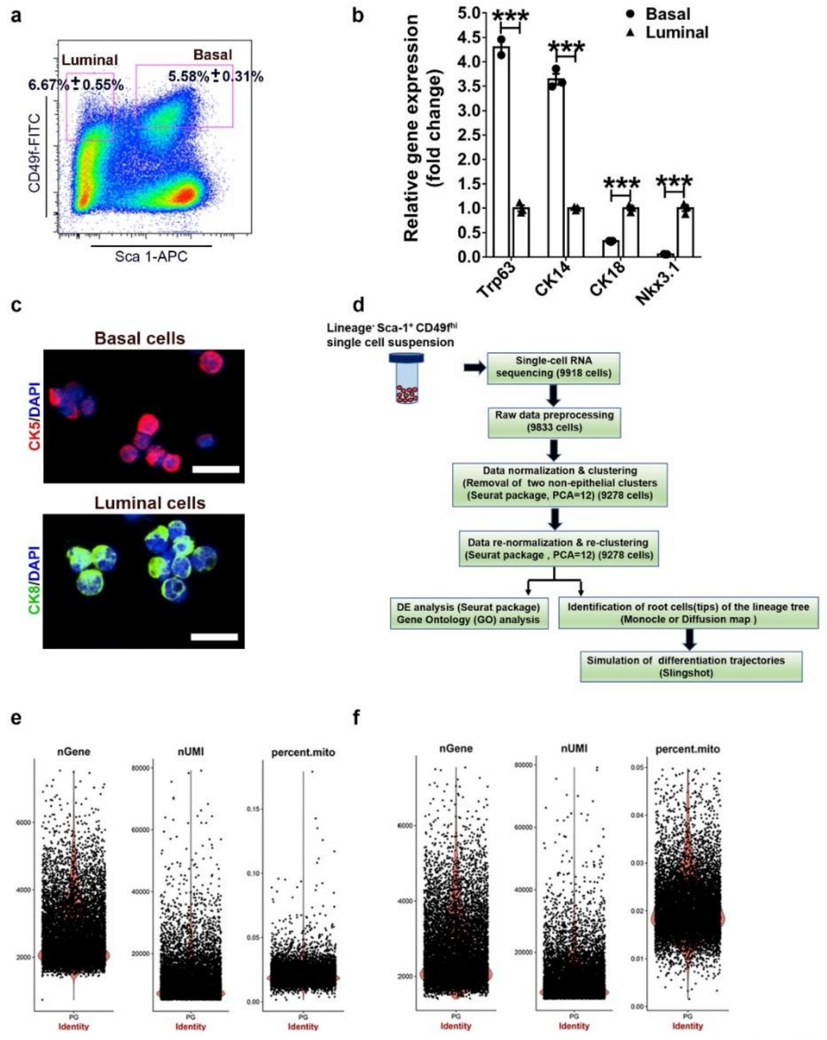

Supplementary Figure 5 | Murine Lineage$^-$ Sca-1$^+$ CD49f^{hi} prostate cells are collected for Single cell RNA-seq.

(a) FACS plots displaying the gating of basal (Lineage$^-$ Sca-1$^+$ CD49f^{hi}） or luminal (Lineage$^-$ Sca 1$^-$ CD49f^{lo}) prostate epithelial cells. The absolute number: basal cells, 17087 ± 2031 and luminal cells, 20427 ± 1025. **(b)** qRT-PCR quantification of basal (Trp63, CK14) and luminal (CK18, Nkx3.1) cell markers in sorted basal and luminal cells. RNA expression levels were normalized to luminal cells (n=3 independent experiments. Data are analyzed by Student's t-test and are presented as mean + s.e.m. ***P<0.001). **(c)** Immunostaining results showing CK5 expression in sorted basal cells and CK8 expression in sorted luminal cells. (**d)** The flow chart for single-cell RNA sequencing data analysis. (**e, f)** Violin plots displaying the distribution of detected numbers of gene, uniqumolecular identifiers (UMI) and percentage of UMIs mapped to mitochondrial genes before (**e**) and after (**f**) removal of low-quality cells (cells with less than 5000 UMIs or less than 1400 genes detected or more than 5% UMI mapped to mitochondria genes were removed).

Supplementary Figure 6

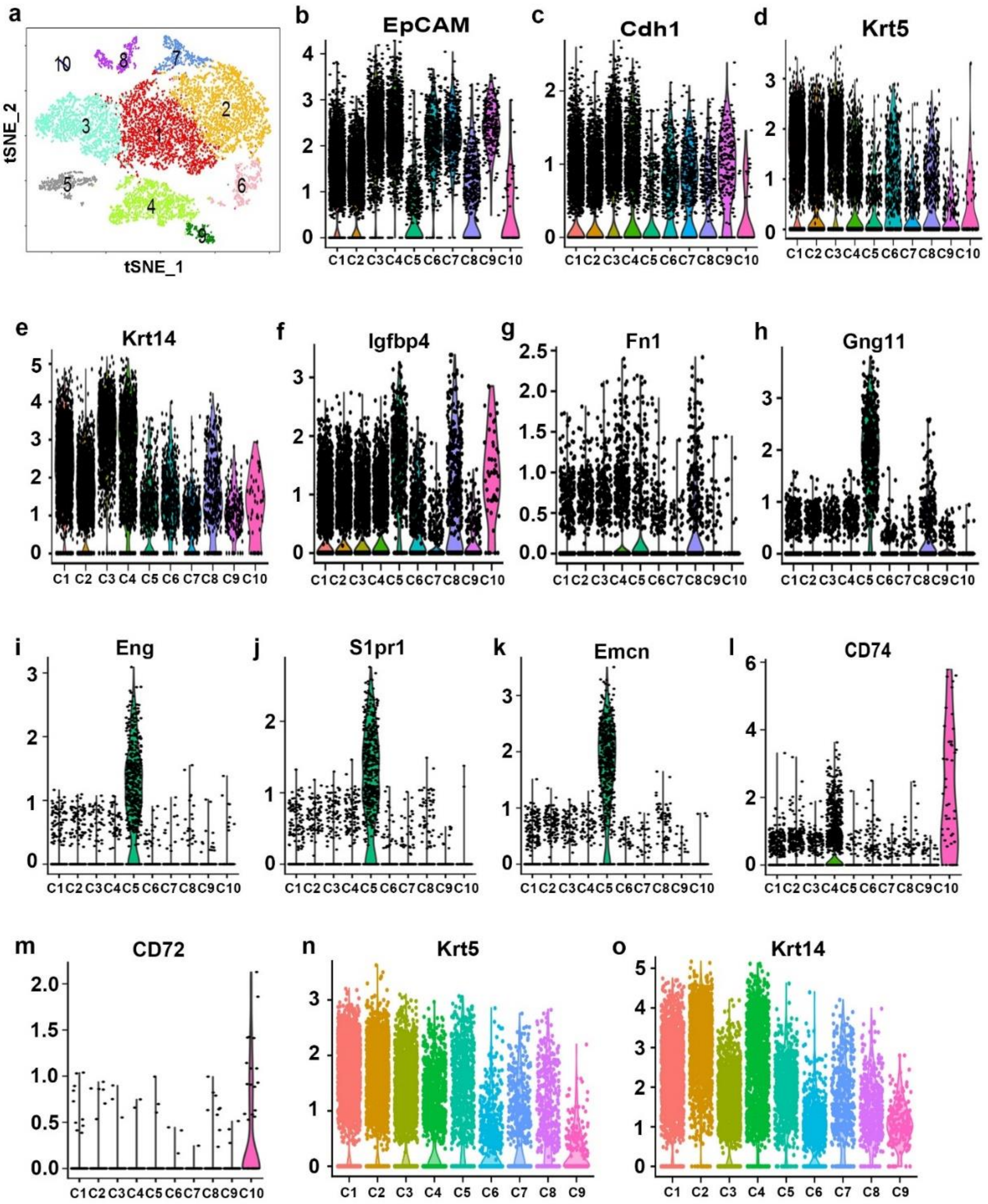

Supplementary Figure 6 | Single cell RNA-seq data pre-processing followed by clustering. (a) A Seurat package and the first 12 principal components were applied to generate 10 different and stable clusters based on differential expressing genes among 9278 mouse prostate basal cells. **(b-m)** Violin plots showing expression difference for epithelial (**b-e**), stromal (**f-h**), endothelial (**i -k**) and immune cell (**l, m**) related genes for the 10 cell clusters before depletion of the non-epithelial cells. **(n, o)** Violin plots showing expression of prostate basal cell markers for the 9 new cell clusters after depleting the non-epithelial cells.

Supplementary Figure 7

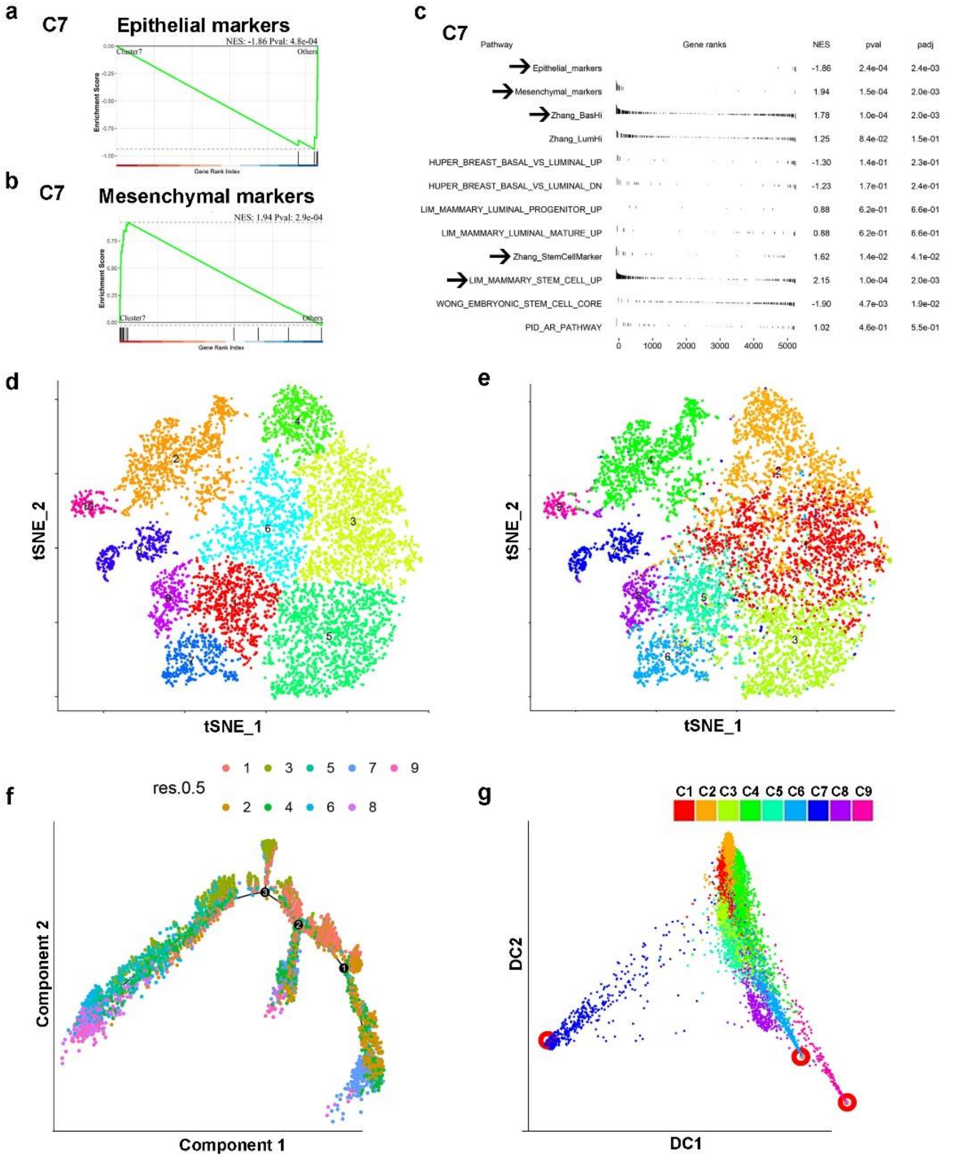

Supplementary Figure 7 | GSEA enrichment and the differentiation trajectory analyses showing that the cell cluster 7 possesses stem cell expression features.

(a, b) GSEA enrichment analyses showing that epithelial cell related markers are downregulated in the cell cluster 7 while mesenchymal cell related markers are enriched in the cell cluster 7. **(c)** GSEA enrichment analyses displaying that previously reported stem cell related expressional profiles were significantly enriched in the cell cluster 7. **(d, e)** t-SNE plots displaying the cell clusters inferred by monocle (**d**) and Seurat (**e**) (components determined for visualization were calculated by monocle). **(f, g)** The differentiation trajectories by monocle (**f**) and diffusion map (**g**) both showed the C7 cluster is one of the tips of the lineage tree (tips were labeled with red circles in (**g**)).

Supplementary Figure 8

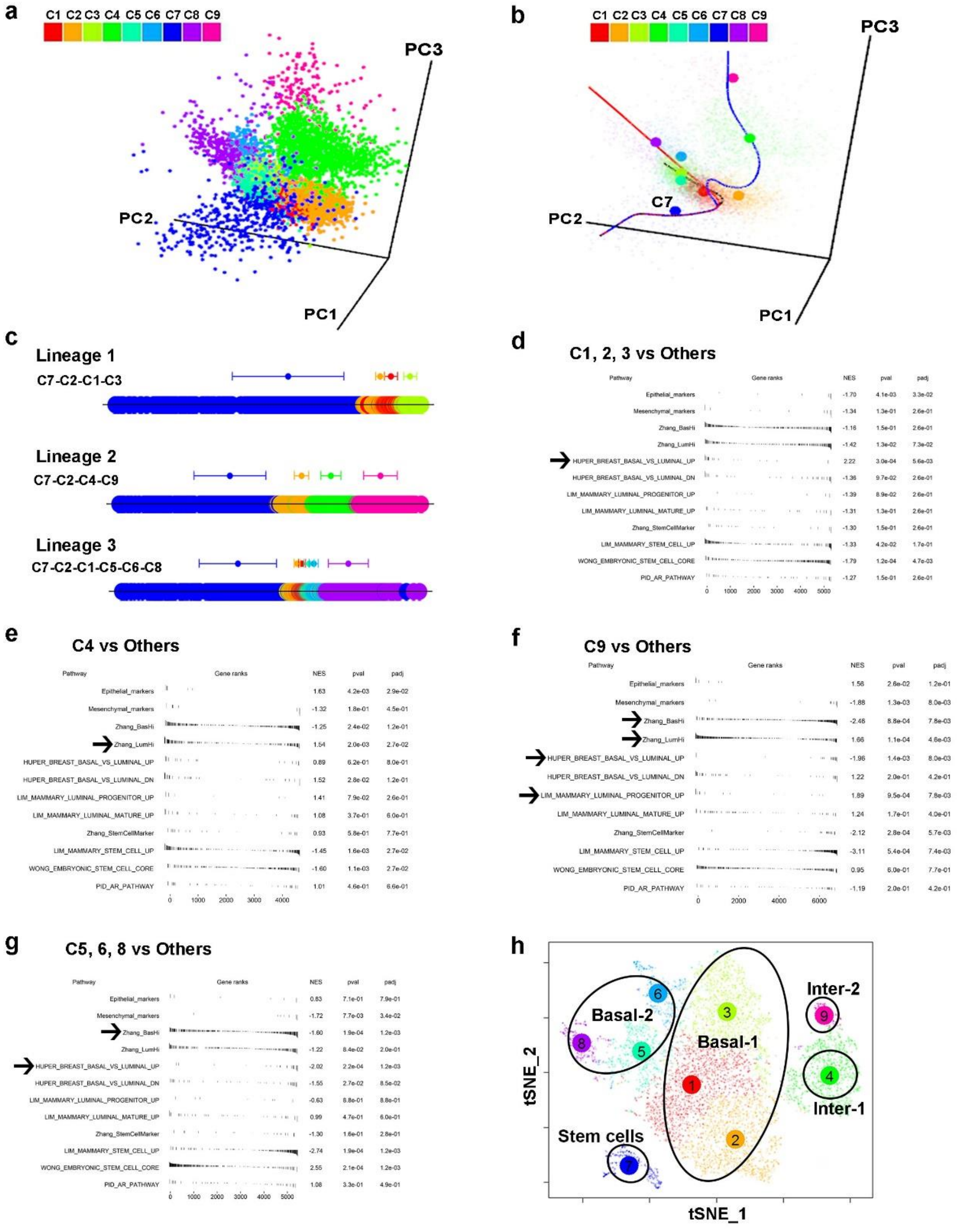

Supplementary Figure 8 | Slingshot predicts three lineage trajectories originated from C7.

(a-c) Slingshot delineate variable cell states and branchpoints in the prostate developmental trajectory. Three differentiation paths originated from C7 are obtained.

(d) In the first differentiation path, cluster 1, 2, 3 express higher levels of basal cell markers, krt5 and krt14, compared to other cell clusters (See Supplementary Fig. 6n, 6o). The number of differentially expressed genes among C1, C2 and C3 are very limited, suggesting they were closed related clusters. C1, C2 and C3 are therefore grouped as a new cluster. GSEA analyses show that the basal cell signature was more highly enriched in C1, 2, 3. C1, 2, 3 is named as “Basal-1” in Supplementary Fig. 8h.

(e-f) In the second differentiation path, cluster 4 and 9 express relative lower levels of basal cell markers (See Supplementary Fig. 6n, 6o). In addition, the GSEA analyses of C4 vs others indicate that the prostate luminal cell signature is upregulated in C4. GSEA analyses of C9 vs others suggest that basal cell signatures are downregulated and the luminal cell signature is upregulated in C9. Yet, the AR pathway signature is not enriched in C4 and C9. Collectively, the C7-C2-C4-C9 path is interpreted to develop into intermediated cells which may further differentiated toward luminal cells. C4 and C9 are named as “Inter-1” and “Inter-2” in Supplementary Fig. 8h.

(g) In the third differentiation path, the number of differentially expressed genes among C5, C6 and C8 are limited. Therefore, they are grouped as a new cluster. GSEA analyses of C5, 6, 8 vs others show that basal cell signatures were downregulated. Considering that C5, 6, 8 indeed expressed a medium level of basal cell marker Krt5 and Krt14 (See Supplementary Fig. 6n, 6o) and that luminal or AR pathway signature are not enriched in C5, 6, 8, it is defined as “Basal-2” in Supplementary Fig. 8h.

(h) t-SNE plot displaying prostate basal cell clusters defined from GSEA analyses.

(Source data are provided as a Source Data file.)

Supplementary Figure 9

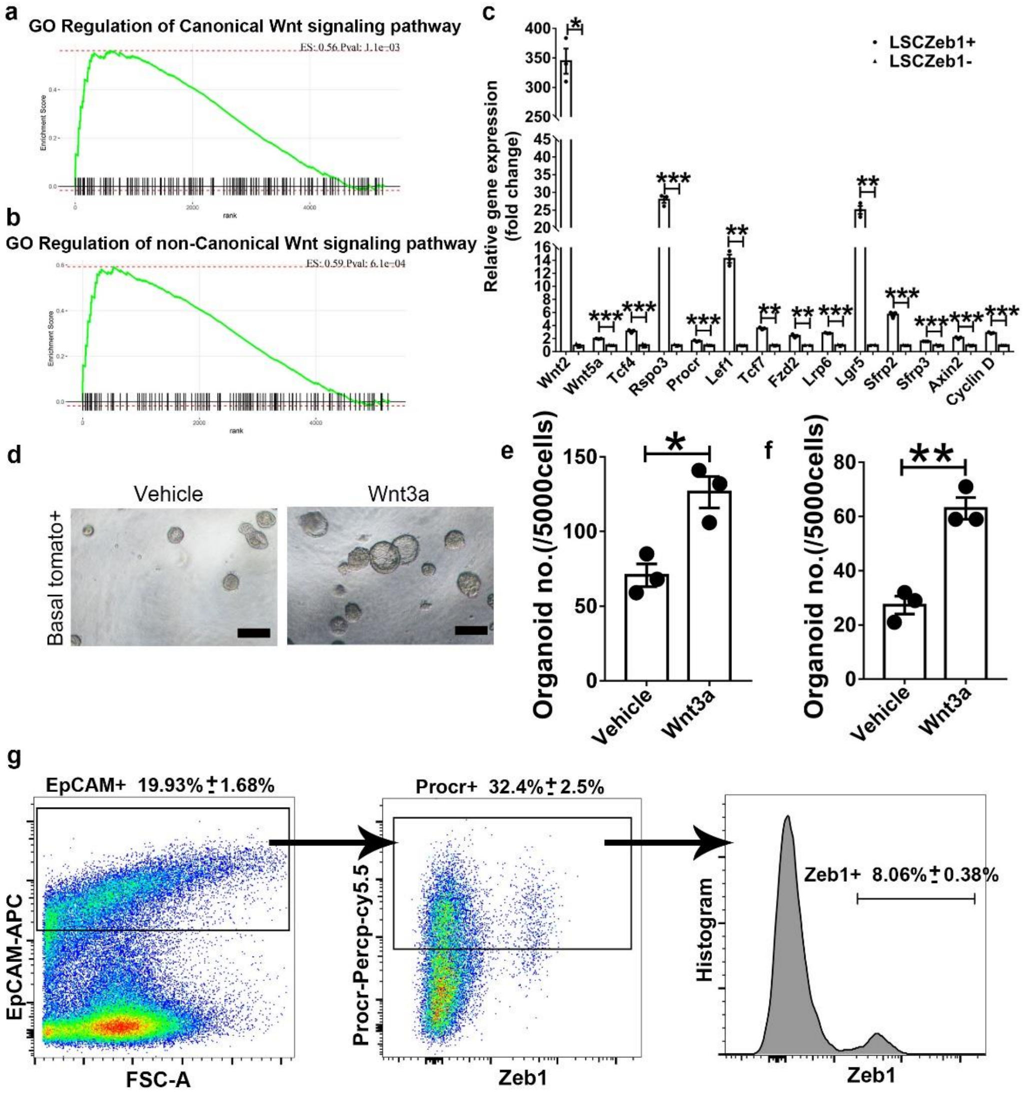

Supplementary Figure 9 | Wnt signaling pathway plays a positive role in the outgrowth of Zeb1$^+$ prostate basal cells.

(a, b) GO enrichment analyses showing that Wnt signaling pathway is positively enriched in the cell cluster 7 compared to other clusters. **(c)** qRT-PCR assays demonstrating that FACS sorted Zeb1$^+$ prostate basal cells significantly highly express components of the Wnt signaling pathway in comparison to Zeb1$^-$ prostate basal cells. **(d-f)** Phase contrast and quantification of the number of and Zeb1$^+$ basal organoids (>100μm) cultured in the medium without and with Wnt3a. (Source data are provided as a Source Data file.) **(g)** FACS analysis of the expression of Zeb1/tdTomato and Procr in EpCAM$^+$ prostate epithelial cells from Zeb1/tdTomato reporter mice. (n=4)

(Scale bars= 200μm. Experiments were repeated for 3 times. Data are analyzed by Student's t-test and are presented as mean + s.e.m. *P<0.05, **P<0.01, ***P<0.001.)

Supplementary Figure 10

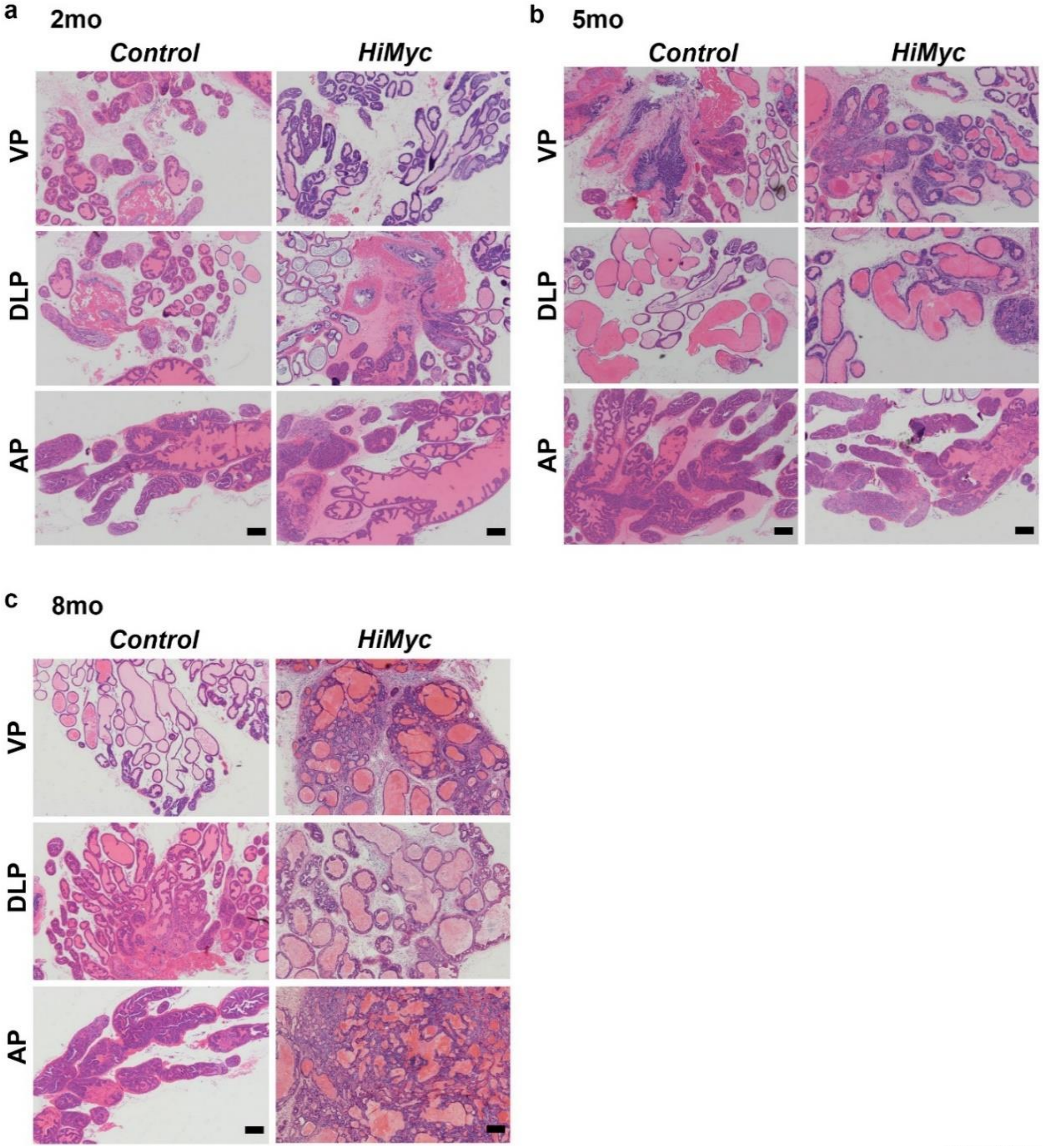

Supplementary Figure 10 | Pathological evaluation and feature characterization for Hi-Myc mice.

(a) H&E staining analysis showed that different prostate lobes, especially ventral and dorsolateral lobes of 2-month old Hi-Myc mice displayed multifocal epithelial hyperplasia.

(b) Low grade murine intraepithelial neoplasia (mPIN) was detected in different prostate lobes from 5-month old Hi-Myc mice.

(c) By 9-month of age, Hi-Myc mice developed high-grade mPIN, featured by increased number of atypical cells with nuclear enlargement, prominent nucleoli and an elevated nuclear to cytoplasmic ratio and microinvasion. (All scale bars= 200μm.)

Supplementary Figure 11

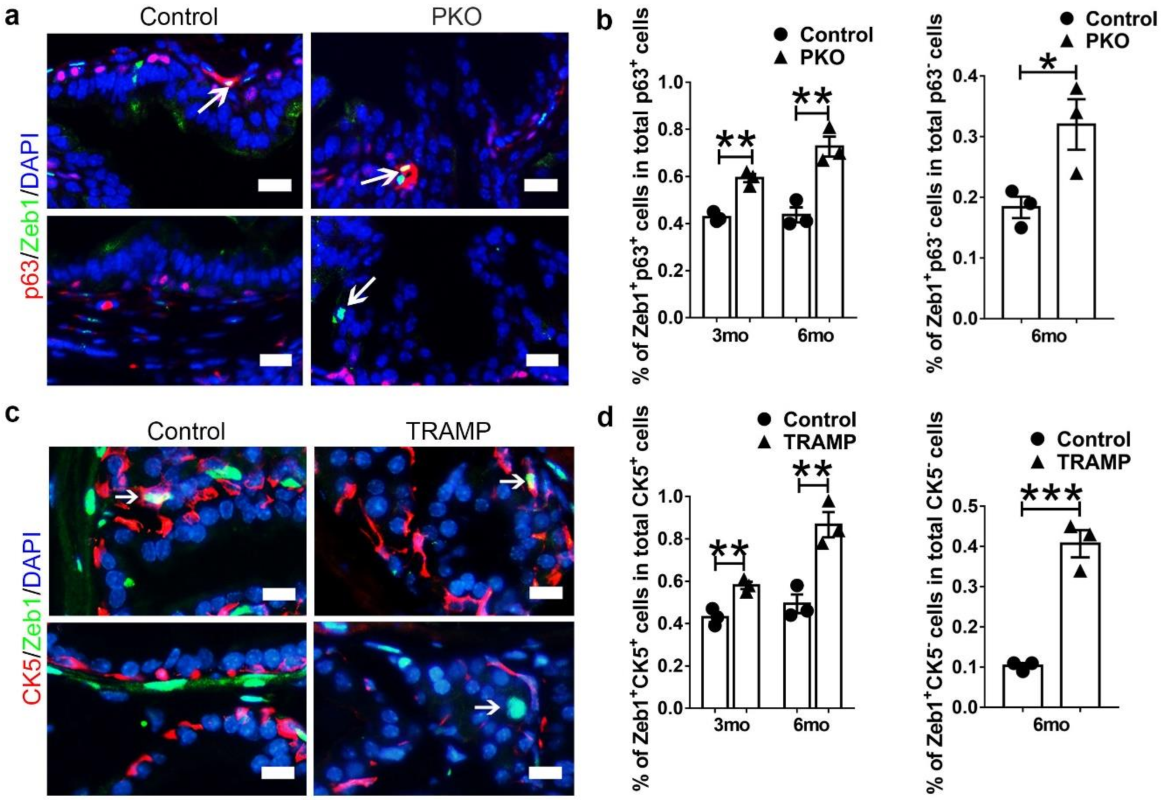

Supplementary Figure 11 | Zeb1^{+} epithelial cells are found in both basal and luminal layer from PKO and TRAMP prostate tumor mouse models.

(a) Images of Zeb1 and p63 co-staining on prostate sections from control or PKO mice (PKO, Pcre;Pten$^{fl/fl}$).

(b) Quantifications of the percentage of Zeb1^{+} p63^{+} basal or Zeb1^{+} p63^{-} luminal cells on control and PKO mouse prostates. (Source data are provided as a Source Data file.)

(c) Images of Zeb1 and CK5 co-staining on prostate sections from control or TRAMP mice.

(d) Quantifications of the percentage of Zeb1^{+} CK5^{+} basal or Zeb1^{+} CK5^{-} luminal cells on control and TRAMP mouse prostates. (Source data are provided as a Source Data file.) (In panel a, c, at least 20 fields per section of 3 sections each mouse prepared from 3 mouse prostates were analyzed. Data are analyzed by Student's t-test and are presented as mean + s.e.m. *P<0.05, **P<0.01, ***P<0.001. All scale bars = 20μm.)

Supplementary Figure 12

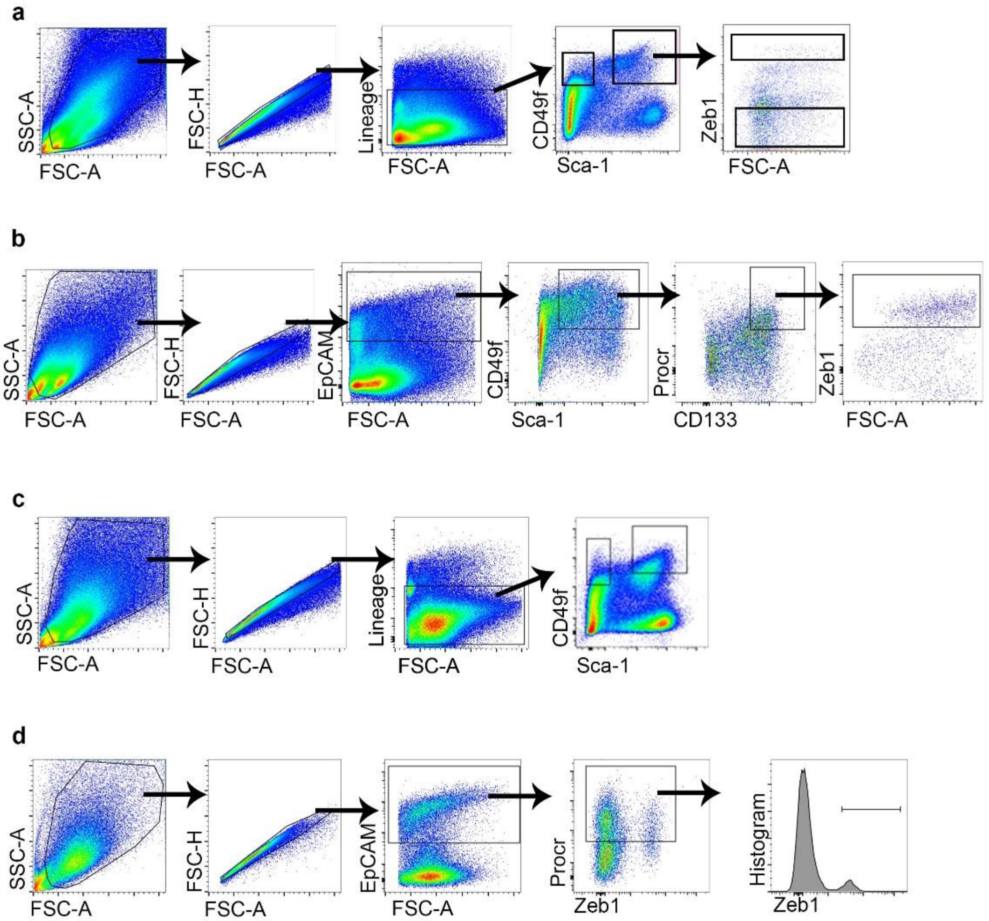

Supplementary Figure 12 | Gating strategies applied for flow cytometry data.

(a) Gating strategy for sorting Lineage$^-$ Sca-1$^+$ CD49^{hi} Zeb1$^+$ cells, Lineage$^-$ Sca-1$^+$ CD49^{hi} Zeb1$^-$ cells and Lineage$^-$ Sca-1$^-$ CD49^{lo} luminal cells from prostate cells. This panel is corresponding to Fig. 2a.

(b) Gating strategy for analyzing the percentage of Zeb1$^+$ cells in Epcam$^+$ Sca-1$^+$ CD49f$^+$ CD133$^+$ Procr$^+$ epithelial cells. This panel is corresponding to Supplementary Figure 4c.

(c) Gating strategy for confirming the purity of sorted prostate basal and luminal prostate epithelial cells. This panel is corresponding to Supplementary Figure 5a.

(d) Gating strategy for obtaining the percentage of Zeb1$^+$ cells in Epcam$^+$ Procr$^+$ prostate epithelial cells. This panel is corresponding to Supplementary Figure 9g.

Supplementary Table 1

Primers used in RT-PCR

Primer ID	Primer 1 (5' to 3')	Primer 2 (3' to 5')
mActin	GGCTGTATTCCCCTCCATCG	CCAGTTGGTAACAATGCCATGT
mZeb1	CCACTGTGGAGGACCAGAAT	CTCGTGAGGCCTCTTACCTG
mtdTomato	ACCAAGCTGGACATCACCTC	GCGCATGAACTCTTTGATGA
mAR	CTGGGAAGGGTCTACCCAC	GGTGCTATGTTAGCGGCCTC
mNkx3-1	ATGCTTAGGGTAGCGGAGC	TGCGGATTGCCTGAGTGTC
mKrt8	TCCATCAGGGTGACTCAGAAA	CCAGCTTCAAGGGGCTCAA
mKrt18	CAGCCAGCGTCTATGCAGG	CTTTCTCGGTCTGGATTCCAC
mKrt5	TCTGCCATCACCCCATCTGT	CCTCCGCCAGAACTGTAGGA
mKrt14	AGCGGCAAGAGTGAGATTTCT	CCTCCAGGTTATTCTCCAGGG
mp63	AGATCCCTGAACAGTTCCGAC	CGACGAGAATCCATGTCAAAGTT
mCD117	GCCACGTCTCAGCCATCTG	GTCGCCAGCTTCAACTATTAACT
mCD133	CTCCCATCAGTGGATAGAGAACT	ATACCCCCTTTTGACGAGGCT
mTrop2	GTGGCTGAGAGTAAATGTGGG	TTGGTGGAATACTTGTCCGCT
mCD166	ATGGCATCTAAGGTGTCCCCT	CTGAGTTGACAGTGTACCATCC
mDlk1	AGTGCGAAACCTGGGTGTC	GCCTCCTTGTTGAAAGTGGTCA
mEpcam	CTGGCGTCTAAATGCTTGGC	CCTTGTCGGTTCTTCGGACTC
mCldn1	TGCCCCAGTGGAAGATTTACT	CTTTGCGAAACGCAGGACAT
mOcln	CTGGATCTATGTACGGCTCACA	TCCACGTAGAGACCAGTACCT
mVim	TCCACACGCACCTACAGTCT	CCGAGGACCGGGTCACATA
mFn1	ATGTGGACCCCTCCTGATAGT	GCCCAGTGATTTCAGCAAAGG
mPrrx1	AAAGAACTTCTCCGTCAGTCACC	CCCACACTTTCGTCTGCTTGT
mPrrx2	CGTGGCACCAAACGAAAGAAG	CGCTCAAATACACGCTCCAG

mSMA	CCCAGACATCAGGGAGTAATGG	TCTATCGGATACTTCAGCGTCA
mZeb2	AAACGTGGTGAACTATGACAACG	CTTGCAGAATCTCGCCACTG
mSnai1	CACACGCTGCCTTGTGTCT	GGTCAGCAAAAGCACGGTT
mSnai2	CAGCGAACTGGACACACACA	ATAGGGCTGTATGCTCCCGAG
mWnt2	CTCGGTGGAATCTGGCTCTG	CACATTGTCACACATCACCCT
mWnt5a	CAACTGGCAGGACTTTCTCAA	CATCTCCGATGCCGGAACT
mTcf4	CGAAAAGTTCCTCCGGGTTTG	CGTAGCCGGGCTGATTCAT
mRspo3	ATGCACTTGCGACTGATTTCT	GCAGCCTTGACTGACATTAGGAT
mProcr	ATGTTGACGAAGTTTCTGCCG	TTGGGAGCCATCGGAGTTACA

Primers used for genotyping

**Primer ID**	**Primer 1 (5' to 3')**	**Primer 2 (3' to 5')**
Zeb1-tdTomato	1. AGGAAGTGGAAGCGGATGAAG	GTAGTGAGCAGGAACCATAGC
	2. TGCCCATAAGTAGAAAGAGCTG	CTCGCCCTTGCTCACCATAG
	3. TGTTCCTGGGGCATGGCACC	GAGATGCCCCAGTGCTTCAG
Zeb1CreERT2	1. GGTTTCCCTGCCACAGCTTGAG	AAGAGTGATAATACTGCTAGCCATGC
	2. CCAAGACTGATGGTACAGTGGAGGT	ACATGTCCATCAGGTTCTTGCGA
	3. GGCCCTTACCAGCCACTTACCTACA	CGACTAGAGCTTGCGGAACCCTT
Zeb1 Knockout	1. AGCACTATTCTCCGCTACTCCAC	
	2. AACCGTGCATCTGCCAGTTTGAG	
	3. ACCGCACCTGGTTTACGACACTC	

Primers used for sgRNA

sgZeb1-1	ACTGCTTATATGTGAGCTAT	
sgZeb1-2	GGAACAACCTGAAGTTGACT	

Supplementary Table 2:

Antibodies used in this paper

Antibodies	Brand	Catalog No.	Dilution
TCF/ZEB1 (D80D3) Rabbit mAb	CST	#3396	1:200
ZEB1(E-20) goat polyclonal	Santa Cruz	sc-10572	1:100
Anti-RFP[EPR18992] rabbit polyclonal	Abcam	ab185921	1:300
Anti-E-cadherin mouse monoclonal	BD	Cat#610181	1:300
Anti-p63 mouse monoclonal	Abcam	Cat#ab735	1:300
Anti-Cytokeratin 5 rabbit monoclonal	Abcam	Cat#ab52635	1:300
Anti-Cytokeratin 8 rabbit monoclonal	Abcam	Cat#ab53280	1:300
Anti-Androgen Receptor rabbit monoclonal	Abcam	Cat#ab133273	1:300
Anti-Sypnaptophysin rabbit polyclonal	Abcam	ab14692	1:200
Anti-probasin rabbit polyclonal	Abcam	ab11575	1:200
Anti-Integrinβ1 clone MB1.2 rat monoclonal	Millpore	MAB1997	1:200
Anti-MHC class I [ER-HR 52] Rat monoclonal	Abcam	ab15681	1:200
Anti-Snai1 (C15D3) rabbit monoclonal	CST	#3879	1:200
Anti-Snai2(Slug) (C19G7) rabbit monoclonal	CST	#9585	1:300
Anti-Twist1/2 rabbit polyclonal	GeneTex	GTX127310	1:200
Anti-Vimentin (D21H3) rabbit monoclonal	CST	#5741	1:200
DAPI	Sigma	D9542	1:1000
Goat anti-Rabbit HRP	CST	#7074	1:1000
Goat anti-Mouse HRP	CST	#7076	1:1000
Biotin Anti-Mouse CD31(clone:390)	eBioscience	13-0311-82	0.5µg per 10^6 cells
Biotin Anti-Mouse CD45.2(clone:104)	eBioscience	13-0454-82	0.5µg per 10^6 cells
Biotin Anti-Mouse TER119(clone:TER-119)	eBioscience	13-5921-85	0.5µg per 10^6 cells
APC Anti-mouse Ly-6A/E (Sca-1)(clone: D7)	eBioscience	17-5981-82	0.5µg per 10^6 cells

FITC Anti-Human/Mouse CD49f(clone:eBioGoH3)	eBioscience	11-0495-82	0.5μg per 10^6 cells
Anti-Mouse CD16/CD32(clone:93)	eBioscience	14-0161-85	0.5μg per 10^6 cells
Alexa Fluor™ 488 donkey anti-mouse IgG(H+L)	Life Technologies	A21202	1:800
Alexa Fluor 594 donkey anti-rabbit IgG(H+L)	Life Technologies	A21207	1:800
Alexa Fluor 594 donkey anti-goat IgG(H+L)	Life Technologies	A11058	1:800
ProLong™ Gold antifade reagent with DAPI	Invitrogen	P36935	1:200
