# Supplementary material for: Identification of a Zeb1 expressing basal stem cell subpopulation in the prostate
Source: Nat Commun. 2020 Feb 5;11:706. doi: 10.1038/s41467-020-14296-y (PMC7002669; doi:10.1038/s41467-020-14296-y)
Supplement: Supplementary file 2 — Reporting Summary [file 41467_2020_14296_MOESM2_ESM.pdf]

nature research

Corresponding author(s): Wei-Qiang Gao and Helen He Zhu

Last updated by author(s): Nov 10,2019

Reporting Summary

Nature Research wishes to improve the reproducibility of the work that we publish. This form provides structure for consistency and transparency in reporting. For further information on Nature Research policies, see Authors & Referees and the Editorial Policy Checklist.

Statistics

For all statistical analyses, confirm that the following items are present in the figure legend, table legend, main text, or Methods section.

n/a	Confirmed	
☐	☒	The exact sample size (*n*) for each experimental group/condition, given as a discrete number and unit of measurement
☐	☒	A statement on whether measurements were taken from distinct samples or whether the same sample was measured repeatedly
☐	☒	The statistical test(s) used AND whether they are one- or two-sided<br>*Only common tests should be described solely by name; describe more complex techniques in the Methods section.*
☒	☐	A description of all covariates tested
☒	☐	A description of any assumptions or corrections, such as tests of normality and adjustment for multiple comparisons
☐	☒	A full description of the statistical parameters including central tendency (e.g. means) or other basic estimates (e.g. regression coefficient) AND variation (e.g. standard deviation) or associated estimates of uncertainty (e.g. confidence intervals)
☐	☒	For null hypothesis testing, the test statistic (e.g. *F*, *t*, *r*) with confidence intervals, effect sizes, degrees of freedom and *P* value noted<br>*Give P values as exact values whenever suitable.*
☒	☐	For Bayesian analysis, information on the choice of priors and Markov chain Monte Carlo settings
☒	☐	For hierarchical and complex designs, identification of the appropriate level for tests and full reporting of outcomes
☒	☐	Estimates of effect sizes (e.g. Cohen's *d*, Pearson's *r*), indicating how they were calculated

Our web collection on statistics for biologists contains articles on many of the points above.

Software and code

Policy information about availability of computer code

Data collection	Codes used for bioinformatic analyses have been deposited in GitHub.<br>Softwares used for bioinformatic analyses have been represented in the manuscript.
Data analysis	Codes used for bioinformatic analyses have been deposited in GitHub.<br>Softwares used for bioinformatic analyses have been represented in the manuscript.

For manuscripts utilizing custom algorithms or software that are central to the research but not yet described in published literature, software must be made available to editors/reviewers. We strongly encourage code deposition in a community repository (e.g. GitHub). See the Nature Research guidelines for submitting code & software for further information.

Data

Policy information about availability of data

All manuscripts must include a data availability statement. This statement should provide the following information, where applicable:

- Accession codes, unique identifiers, or web links for publicly available datasets
- A list of figures that have associated raw data
- A description of any restrictions on data availability

The single cell RNA-seq raw data used for this study are available at the GEO web with the accession number GSE111429. Codes used are deposited to the website https://github.com/HelenHeZhu/StemCell. All other data is available in the main text or the supplementary materials. The source data underlying Figs 1b, 1h-j, 2e-i, 3p, 4i, 5d, 5h, 6e-h, 7 and Supplementary Figs 1c, 2, 3f, 9e,f, 11 are provided as a Source Data file.

Field-specific reporting

Please select the one below that is the best fit for your research. If you are not sure, read the appropriate sections before making your selection.

☒ Life sciences ☐ Behavioural & social sciences ☐ Ecological, evolutionary & environmental sciences

For a reference copy of the document with all sections, see nature.com/documents/nr-reporting-summary-flat.pdf

Life sciences study design

All studies must disclose on these points even when the disclosure is negative.

Sample size	The sample sizes for the each experiments were described in the relevant figure legends of the paper
Data exclusions	No sample was excluded in the analysis.
Replication	The number of animals for each experiment is at least 3. Three individual experiments were performed in the RT-PCR assay. All attempts at replication were successful.
Randomization	The animals were randomizedly chosen for treatment to ensure reliance of each experiment.
Blinding	The animals were randomizedly chosen for treatment to ensure reliance of each experiment.

Reporting for specific materials, systems and methods

We require information from authors about some types of materials, experimental systems and methods used in many studies. Here, indicate whether each material, system or method listed is relevant to your study. If you are not sure if a list item applies to your research, read the appropriate section before selecting a response.

Materials & experimental systems

n/a	Involved in the study	
☐	☒	Antibodies
☒	☐	Eukaryotic cell lines
☒	☐	Palaeontology
☐	☒	Animals and other organisms
☐	☒	Human research participants
☒	☐	Clinical data

Methods

n/a	Involved in the study	
☒	☐	ChIP-seq
☐	☒	Flow cytometry
☒	☐	MRI-based neuroimaging

Antibodies

Antibodies used

TCF/ZEB1 (D80D3) Rabbit mAb CST #3396
ZEB1(E-20) goat polyclonal Santa Cruz sc-10572
Anti-RFP[EPR18992] rabbit polyclonal Abcam ab185921
Anti-E-cadherin mouse monoclonal BD Cat#610181
Anti-p63 mouse monoclonal Abcam Cat#ab735
Anti-Cytokeratin 5 rabbit monoclonal Abcam Cat#ab52635
Anti-Cytokeratin 8 rabbit monoclonal Abcam Cat#ab53280
Anti-Androgen Receptor rabbit monoclonal Abcam Cat#ab133273
Anti-Sypnaptophysin rabbit polyclonal Abcam ab14692
Anti-probasin rabbit polyclonal Abcam ab11575
Anti-Integrinβ1 clone MB1.2 rat monoclonal Millpore MAB1997
Anti-MHC class I [ER-HR 52] Rat monoclonal Abcam ab15681
Anti-Snai1 (C15D3) rabbit monoclonal CST #3879
Anti-Snai2(Slug) (C19G7) rabbit monoclonal CST #9585
Anti-Twist1/2 rabbit polyclonal GeneTex GTX127310
Anti-Vimentin (D21H3) rabbit monoclonal CST #5741
DAPI Sigma D9542
Goat anti-Rabbit HRP CST #7074
Goat anti-Mouse HRP CST #7076
Biotin Anti-Mouse CD31(clone:390) eBioscience 13-0311-82
Biotin Anti-Mouse CD45.2(clone:104) eBioscience 13-0454-82
Biotin Anti-Mouse TER119(clone:TER-119) eBioscience 13-5921-85
APC Anti-mouse Ly-6A/E (Sca-1)(clone: D7) eBioscience 17-5981-82
FITC Anti-Human/Mouse CD49f(clone:eBioGoH3) eBioscience 11-0495-82
Anti-Mouse CD16/CD32(clone:93) eBioscience 14-0161-85

Alexa FluorTM 488 donkey anti-mouse IgG(H+L) Life Technologies A21202
Alexa Fluor 594 donkey anti-rabbit IgG(H+L) Life Technologies A21207
Alexa Fluor 594 donkey anti-goat IgG(H+L) Life Technologies A11058
ProLongTM Gold antifade reagent with DAPI Invitrogen P36935

Validation

TCF/ZEB1 (D80D3) Rabbit mAb CST #3396 validation reference PMID:17486063
ZEB1(E-20) goat polyclonal Santa Cruz sc-10572 validation based on manufacturer's data sheet
Anti-RFP[EPR18992] rabbit polyclonal Abcam ab185921 validation based on manufacturer's data sheet
Anti-E-cadherin mouse monoclonal BD Cat#610181 validation reference PMID:25163637
Anti-p63 mouse monoclonal Abcam Cat#ab735 validation reference PMID: 19956733
Anti-Cytokeratin 5 rabbit monoclonal Abcam Cat#ab52635 validation reference PMID: 24796293
Anti-Cytokeratin 8 rabbit monoclonal Abcam Cat#ab53280 validation reference PMID: 25176651
Anti-Androgen Receptor rabbit monoclonal Abcam Cat#ab133273 validation reference PMID:25163637
Anti-Sypnaptophysin rabbit polyclonal Abcam ab14692 validation reference PMID:27775035
Anti-probasin rabbit polyclonal Abcam ab11575 validation reference PMID:28348210
Anti-Integrinβ1 clone MB1.2 rat monoclonal Millpore MAB1997 validation reference PMID:18946470
Anti-MHC class I [ER-HR 52] Rat monoclonal Abcam ab15681 validation reference PMID:20007464
Anti-Snai1 (C15D3) rabbit monoclonal CST #3879 validation reference PMID:30540935
Anti-Snai2(Slug) (C19G7) rabbit monoclonal CST #9585 validation reference PMID:29158396
Anti-Twist1/2 rabbit polyclonal GeneTex GTX127310 validation based on manufacturer's data sheet
Anti-Vimentin (D21H3) rabbit monoclonal CST #5741 validation reference PMID:29988032
Goat anti-Rabbit HRP CST #7074 validation reference PMID:31260412
Goat anti-Mouse HRP CST #7076 validation reference PMID:31409799
Biotin Anti-Mouse CD31(clone:390) eBioscience 13-0311-82 validation reference PMID:30181538
Biotin Anti-Mouse CD45.2(clone:104) eBioscience 13-0454-82 validation reference PMID: 24487275
Biotin Anti-Mouse TER119(clone:TER-119) eBioscience 13-5921-85 validation reference PMID: 29934585
APC Anti-mouse Ly-6A/E (Sca-1)(clone: D7) eBioscience 17-5981-82 validation reference PMID: 30187863
FITC Anti-Human/Mouse CD49f(clone:eBioGoH3) eBioscience 11-0495-82 validation reference PMID: 29045388
Anti-Mouse CD16/CD32(clone:93) eBioscience 14-0161-85 validation reference PMID:27278624
Alexa FluorTM 488 donkey anti-mouse IgG(H+L) Life Technologies A21202 validation reference PMID: 28408404
Alexa Fluor 594 donkey anti-rabbit IgG(H+L) Life Technologies A21207 validation reference PMID:25571975
Alexa Fluor 594 donkey anti-goat IgG(H+L) Life Technologies A11058 validation reference PMID: 28198371

Animals and other organisms

Policy information about studies involving animals; ARRIVE guidelines recommended for reporting animal research

Laboratory animals

C57BL/6 mice,both male and female, were used in this study, and described in the paper for detailes.

Wild animals

The study did not involved wild animals.

Field-collected samples

The study did not involve the samples collected from the field

Ethics oversight

All mice were maintained and bred in the pathogen-free facility at Renji Hospital. Mouse experimental protocols were approved by the Renji Hospital Animal Care and Use Committee.

Note that full information on the approval of the study protocol must also be provided in the manuscript.

Human research participants

Policy information about studies involving human research participants

Population characteristics

Freshly dissected human prostate specimens (non-BPH, BPH and prostate cancer specimens) were obtained from the department of Urology at Ren Ji Hospital with informed consent from patients. All the characteristics are listed in Supplementary Table X.

Recruitment

All patients information and samples were collected based on clinical requirements for diagnosis.

Ethics oversight

All Human sample experiments were conducted according to the ethical regulations of Ren Ji Hospital. Human sample collection and handling protocols were approved by the Ren Ji Ethics committee.

Note that full information on the approval of the study protocol must also be provided in the manuscript.

Flow Cytometry

Plots

Confirm that:

☒ The axis labels state the marker and fluorochrome used (e.g. CD4-FITC).

☒ The axis scales are clearly visible. Include numbers along axes only for bottom left plot of group (a 'group' is an analysis of identical markers).

☒ All plots are contour plots with outliers or pseudocolor plots.

☒ A numerical value for number of cells or percentage (with statistics) is provided.

Methodology

Sample preparation	The protocols for sample preparations were represented in the methods part of the main manuscript.
Instrument	BD Accuri C6 or FACSAria III flow cytometer
Software	FolwJo software is used for FACS data analyses.
Cell population abundance	Cells were sorted based on the purity.
Gating strategy	Gating strategies were based on the isotype control, single color controls and fluorescence minus one controls.

☒ Tick this box to confirm that a figure exemplifying the gating strategy is provided in the Supplementary Information.
